# Translational barriers to phage endolysin deployment for antimicrobial resistance control in Africa

**DOI:** 10.3389/frabi.2026.1815611

**Published:** 2026-04-13

**Authors:** Anayochukwu Chibuike Ngene, Michael Ndubuisi Umeh, Maryjoy Chioma Ibeh, Ekene Samuel Odo, Chinedu Godspower Ohaegbu

**Affiliations:** 1 Department of Microbiology, College of Natural Sciences, Michael Okpara University of Agriculture, Umudike, Nigeria; 2 Department of Plant Health Management, College of Crop and Soil Sciences, Michael Okpara University of Agriculture, Umudike, Nigeria

**Keywords:** AMR policy, biotechnology capacity, enzybiotics, phage therapy, phage-derived antimicrobials, translational research

## Abstract

Antimicrobial resistance (AMR) poses a rapidly escalating threat to public health, food security, and environmental sustainability across Africa, where limited diagnostic capacity, unregulated antibiotic use, and weak pharmaceutical pipelines exacerbate treatment failures. Bacteriophage-derived endolysins have emerged globally as promising next-generation antimicrobials due to their rapid bacteriolytic activity, low resistance potential, and modular engineering flexibility. Despite significant advances in endolysin discovery, engineering, and clinical translation worldwide, their deployment in Africa remains negligible. This disparity raises a critical question: *why have phage endolysins not progressed beyond early-stage research in Africa despite their relevance to the continent’s AMR burden?* This review provides a critical analysis of the translational barriers hindering the development, adoption, and commercialization of phage endolysins for AMR control in Africa. Drawing on published African and global literature, we identify interconnected scientific, infrastructural, regulatory, economic, and policy-related constraints that limit progress from laboratory discovery to real-world application. Key barriers include limited genomic and protein engineering capacity, absence of regional phage and endolysin repositories, inadequate biosafety and regulatory frameworks for biologics, dependence on imported reagents and expression systems, and weak integration of lysin technologies into national AMR and One Health action plans. We further highlight a mismatch between globally prioritized lysin targets and Africa’s dominant clinical, agricultural, and environmental pathogens. By reframing the African endolysin landscape through a translational failure lens, this review moves beyond descriptive summaries to propose actionable pathways for overcoming these barriers. We outline strategic priorities for capacity building, regulatory harmonization, funding mechanisms, and regional collaboration necessary to enable Africa’s participation in the global endolysin pipeline. Addressing these translational bottlenecks is essential for ensuring equitable access to lysin-based antimicrobials and for positioning Africa as an active contributor to next-generation AMR solutions rather than a passive end-user.

## Graphical Abstract

**Figure:**
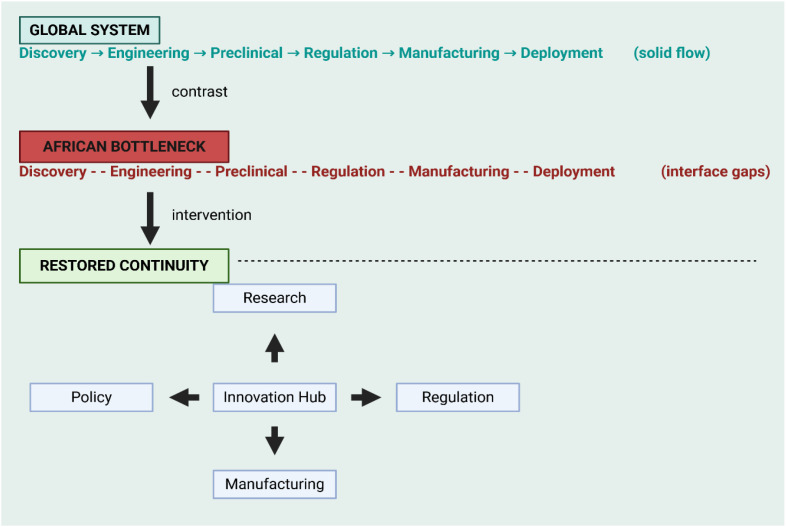

## 1 Introduction

Antimicrobial resistance in Africa is not primarily a consequence of scientific ignorance, but of failed translation (the persistent inability to convert existing antimicrobial innovations into accessible, deployable solutions within fragile health, agricultural, and environmental systems) (Abia and Essack, 2023). While global antimicrobial research pipelines continue to generate advanced biologics and next-generation therapeutics, African countries remain disproportionately burdened by multidrug-resistant (MDR) infections with limited access to these innovations. This widening translational gap has become one of the defining challenges of AMR control on the continent (Ngene and Umar, 2025).

Among emerging antimicrobial technologies, bacteriophage-derived endolysins have attracted significant global attention as precision antimicrobials capable of rapidly lysing bacterial pathogens, disrupting biofilms, and circumventing many classical resistance mechanisms (Khan et al., 2023). Over the past two decades, extensive progress has been made in endolysin discovery, structural characterization, protein engineering, formulation, and early-stage clinical evaluation, particularly in Europe, North America, and parts of Asia (Ho et al., 2022). Several lysin candidates have advanced to preclinical and clinical development, underscoring their therapeutic potential as alternatives or adjuncts to conventional antibiotics (Antonova et al., 2024).

Paradoxically, despite the relevance of endolysins to Africa’s AMR landscape (characterized by high burdens of bacterial infections, widespread antibiotic misuse, and expanding zoonotic and environmental reservoirs), endolysin research and deployment in Africa remain extremely limited (Murray et al., 2021). To date, only a small number of African-based studies have progressed beyond basic phage isolation or in silico analyses, with virtually no locally developed endolysin candidates reaching advanced preclinical testing, product formulation, or regulatory consideration. This disconnect raises a fundamental but underexplored question: why have phage endolysins failed to translate in Africa despite their global scientific maturity and contextual relevance?

Most existing reviews on endolysins focus on molecular mechanisms, enzymatic classification, engineering strategies, or therapeutic applications, often presenting Africa as a passive setting where such technologies could be applied in the future (Tesema, 2025). While these contributions are valuable, they largely overlook the structural, institutional, and systemic constraints that prevent endolysin technologies from moving from proof-of-concept to real-world implementation in African contexts. As a result, the African endolysin landscape is frequently framed as a problem of “limited data” rather than as a predictable outcome of translational bottlenecks spanning infrastructure, regulation, funding, intellectual property, and capacity development.

This review departs deliberately from descriptive summaries of endolysin biology and instead adopts a translational barrier–focused perspective. We critically examine the scientific, infrastructural, regulatory, economic, and policy-related factors that collectively hinder the development, adoption, and commercialization of phage endolysins for AMR control in Africa. Particular attention is given to (i) limitations in genomic sequencing and protein engineering capacity, (ii) the absence of regional phage and endolysin repositories, (iii) underdeveloped biosafety and regulatory pathways for biologic antimicrobials, (iv) dependence on imported reagents and foreign intellectual property, and (v) weak integration of lysin technologies into national AMR and One Health action plans.

By reframing Africa’s limited engagement with endolysin technology as a problem of translational readiness constrained by identifiable barriers, this review provides a new conceptual lens through which African AMR innovation gaps can be understood and addressed. Rather than asking whether endolysins are scientifically promising, we ask what must change (structurally, institutionally, and politically) for Africa to participate meaningfully in the global endolysin pipeline. Addressing these barriers is essential not only for equitable access to next-generation antimicrobials but also for positioning Africa as an active contributor to AMR solutions rather than a downstream consumer of externally developed technologies.

This review is intended for researchers, regulators, and policy actors working at the intersection of antimicrobial innovation, biologics regulation, and One Health systems in low- and middle-income settings.

## 2 The translational landscape of phage endolysins

The translational trajectory of bacteriophage-derived endolysins reflects one of the most advanced examples of enzyme-based antimicrobial development within modern biotechnology (Ho et al., 2022; Khan et al., 2023). Unlike conventional antibiotics, which emerged largely from empirical screening of small molecules, endolysins are products of rational biological design, informed by genomics, structural biology, and protein engineering (Wojciechowska, 2025; Shemyakin et al., 2020). Over the past two decades, substantial global investment has transformed endolysins from phage lytic enzymes into clinically tractable antimicrobial candidates. Understanding this global development landscape is essential for contextualizing Africa’s limited participation and for identifying where translational discontinuities occur.

### 2.1 Discovery, engineering, and the global development pipeline

Endolysin discovery initially relied on the isolation of lytic bacteriophages followed by biochemical characterization of their cell wall–degrading enzymes (Donovan, 2007; Linden et al., 2015). However, advances in whole-genome sequencing and metagenomics have fundamentally reshaped this process. Contemporary discovery pipelines now routinely mine phage genomes, prophage regions within bacterial chromosomes, and environmental metagenomes to identify putative lysin genes without the need for culturable phages (Schmitz et al., 2010; Andrade-Martinez et al., 2022). This shift has enabled rapid expansion of lysin libraries targeting a wide range of bacterial taxa.

Structurally, most endolysins exhibit a modular architecture composed of enzymatically active domains (EADs) responsible for peptidoglycan cleavage and cell wall binding domains (CBDs) that confer host specificity (Aitken et al., 2025). This modularity has become a cornerstone of lysin engineering, allowing domains to be recombined, truncated, or optimized to enhance bactericidal activity, broaden host range, or improve stability. Engineering strategies such as domain shuffling, fusion with membrane-disrupting peptides, and charge modification have been particularly effective in extending lysin activity against Gram-negative bacteria, historically considered less susceptible due to the outer membrane barrier (Heselpoth et al., 2019).

Beyond molecular optimization, the global development pipeline has increasingly emphasized translational readiness. Engineered lysins are now routinely subjected to standardized *in vitro* efficacy testing, biofilm disruption assays, synergy studies with antibiotics, and toxicity screening in mammalian systems (De Maesschalck et al., 2020). Several candidates have progressed into advanced preclinical development and early-phase clinical evaluation, particularly for infections caused by *Staphylococcus aureus*, *Streptococcus* spp., and *Enterococcus* spp (Taati Moghadam et al., 2025; Cui et al., 2025). Parallel advances in recombinant protein expression, purification scalability, and formulation science have further strengthened the clinical feasibility of lysin-based therapeutics.

Importantly, the global pipeline benefits from a tightly integrated ecosystem linking academic laboratories, biotechnology companies, contract manufacturing organizations, and regulatory agencies. Dedicated phage and lysin repositories, shared genomic databases, and well-defined regulatory pathways for biologics have collectively enabled relatively seamless progression from discovery to clinical translation. This mature translational infrastructure stands in stark contrast to the fragmented and early-stage nature of endolysin research in most African settings, where discovery efforts rarely extend beyond genomic identification or preliminary activity assays.

#### 2.1.1 Success stories and global efforts in bridging development gaps for phage-derived endolysins

Despite notable setbacks—such as the early termination of ContraFect’s exebacase (CF-301) Phase 3 program and subsequent company bankruptcy—phage-derived endolysins have produced tangible success stories and sustained international momentum that are actively closing gaps across the full pipeline, from initial discovery and protein engineering through GMP-scale manufacturing to practical clinical deployment (Vander Elst, 2026).

A flagship commercial achievement is Micreos Human Health (Netherlands) successfully bringing Staphefekt SA.100 (marketed as Gladskin) to market in 2013—the world’s first phage-derived endolysin product available over-the-counter in Europe. This topical formulation selectively eliminates *S. aureus* (including MRSA) while preserving beneficial skin commensals, reducing colonization, and improving symptoms in atopic dermatitis, acne, and eczema, as evidenced by real-world case studies and customer/physician feedback. It has bridged manufacturing (stable, ready-to-use cream and gel formats produced recombinantly) and deployment phases without needing a prescription, providing immediate patient access and microbiome-modulating benefits (Micreos Human Health, 2020; Vander Elst, 2026).

Building on this foundation, Micreos’ optimized variant XZ.700—an engineered endolysin sharing the same core domains but with improved linker design—is currently in an ongoing Phase I/IIa trial as the first pharmaceutical-grade endolysin evaluated topically for mild-to-moderate atopic dermatitis (monotherapy, no antibiotics). Preclinical data further show accelerated wound healing, biofilm disruption on medical implants, and potential utility in cutaneous T-cell lymphoma by controlling *S. aureus* without disrupting the skin microbiome (Micreos Human Health, 2020; Vander Elst, 2026).

In systemic development, iNtRON Biotechnology (South Korea) has advanced SAL200 (tonabacase) to “Phase 2b-ready” status (iNtRON Biotechnology, 2026) with full U.S. FDA Investigational New Drug (IND) clearance after completing Phase I (safety and pharmacokinetics in healthy volunteers) and a partial Phase IIa trial in *S. aureus* bacteremia patients. Recent highlights include 100% sterilization of cardiac valves and major organs in a rabbit endocarditis model and active global out-licensing discussions at the 2026 AMR Conference in Switzerland with partners such as Basilea Pharmaceutica and other international pharma/biotech firms. These efforts directly address partnership, funding, and deployment gaps for intravenous adjunctive use (iNtRON Biotechnology, 2026).

Complementary global programs further illustrate progress:

- P128 (GangaGen, India) — completed Phase I/II nasal decolonization trials (standalone spray, no antibiotics) in healthy volunteers and chronic kidney disease patients.
- HY-133 (HYpharm, Germany) — ongoing Phase I for nasal decolonization (hydrogel/ointment formats).
- LMN-201 (Lumen Bioscience, USA) — Phase II for recurrent *C. difficile colitis* (engineered cocktail with standard-of-care antibiotics).

These initiatives, regularly featured in WHO antibacterial pipeline reviews, leverage high-throughput recombinant expression systems for cost-effective, GMP-compliant manufacturing and employ targeted formulations (topical, nasal, intravenous, gastrointestinal) to overcome stability and delivery challenges. Protein engineering advances—domain swapping, chimeric fusions (e.g., Artilysins for Gram-negative expansion), and catalytic-site optimization—continue to fill mechanistic gaps by enhancing lytic speed, biofilm penetration, serum synergy, and resistance refractoriness (Aitken et al., 2025; Vander Elst, 2026).

Collectively, these examples demonstrate a resilient global ecosystem (spanning Europe, Asia, and North America) that is steadily translating phage-derived endolysins from laboratory discovery into safe, effective tools against resistant pathogens, with topical/cosmetic deployment already achieved and systemic/combination strategies advancing rapidly toward broader regulatory approval and real-world integration.

### 2.2 Advantages of endolysins over conventional antibiotics

The growing interest in endolysins as antimicrobial agents is driven by several intrinsic advantages that directly address limitations associated with conventional antibiotics. Chief among these is their rapid and targeted bacteriolytic activity. Endolysins act enzymatically to cleave essential bonds within the bacterial peptidoglycan, leading to immediate osmotic lysis upon contact with susceptible cells (Grishin et al., 2020). This mechanism bypasses many metabolic pathways commonly exploited by antibiotics, reducing the likelihood of pre-existing resistance.

Unlike broad-spectrum antibiotics that exert extensive selective pressure across microbial communities, endolysins typically display narrow-spectrum specificity. This precision enables effective pathogen elimination while preserving commensal microbiota, an increasingly recognized determinant of host immunity and infection resilience. Reduced microbiome disruption also lowers the risk of secondary infections and limits the ecological drivers of antimicrobial resistance dissemination (Murray et al., 2021).

Another key advantage lies in the low propensity for resistance development. Because endolysins target highly conserved and structurally constrained components of the bacterial cell wall, mutations conferring resistance are often deleterious to bacterial viability (Rahman et al., 2021). While adaptive responses such as cell wall modification have been reported under experimental conditions, stable clinical resistance to lysins remains exceedingly rare compared to conventional antibiotics (Bhagwat et al., 2020; Oechslin et al., 2022).

Endolysins also demonstrate strong activity against biofilm-associated bacteria, a major contributor to chronic and device-related infections (Liu B. et al., 2023; Liu B. et al., 2023). Their enzymatic action enables penetration and degradation of biofilm matrices, either as standalone agents or in synergistic combination with antibiotics. This property has positioned lysins as attractive adjunct therapies capable of restoring antibiotic susceptibility in multidrug-resistant infections.

From a translational standpoint, endolysins offer additional advantages as biologic therapeutics. Their defined molecular structures facilitate rational optimization, predictable pharmacodynamics, and targeted delivery strategies. Furthermore, lysins can be engineered for topical, systemic, veterinary, or environmental applications, aligning well with One Health approaches to AMR control.

Collectively, these attributes explain the accelerating global interest in endolysin-based antimicrobials and their advancement within international development pipelines. However, these same advantages also highlight the inequity of access and participation, as regions most burdened by antimicrobial resistance (including much of Africa) remain largely excluded from the scientific, infrastructural, and regulatory systems required to translate endolysins from promising molecules into deployable solutions.

## 3 Translational barriers in the African context

**Figure 1:**
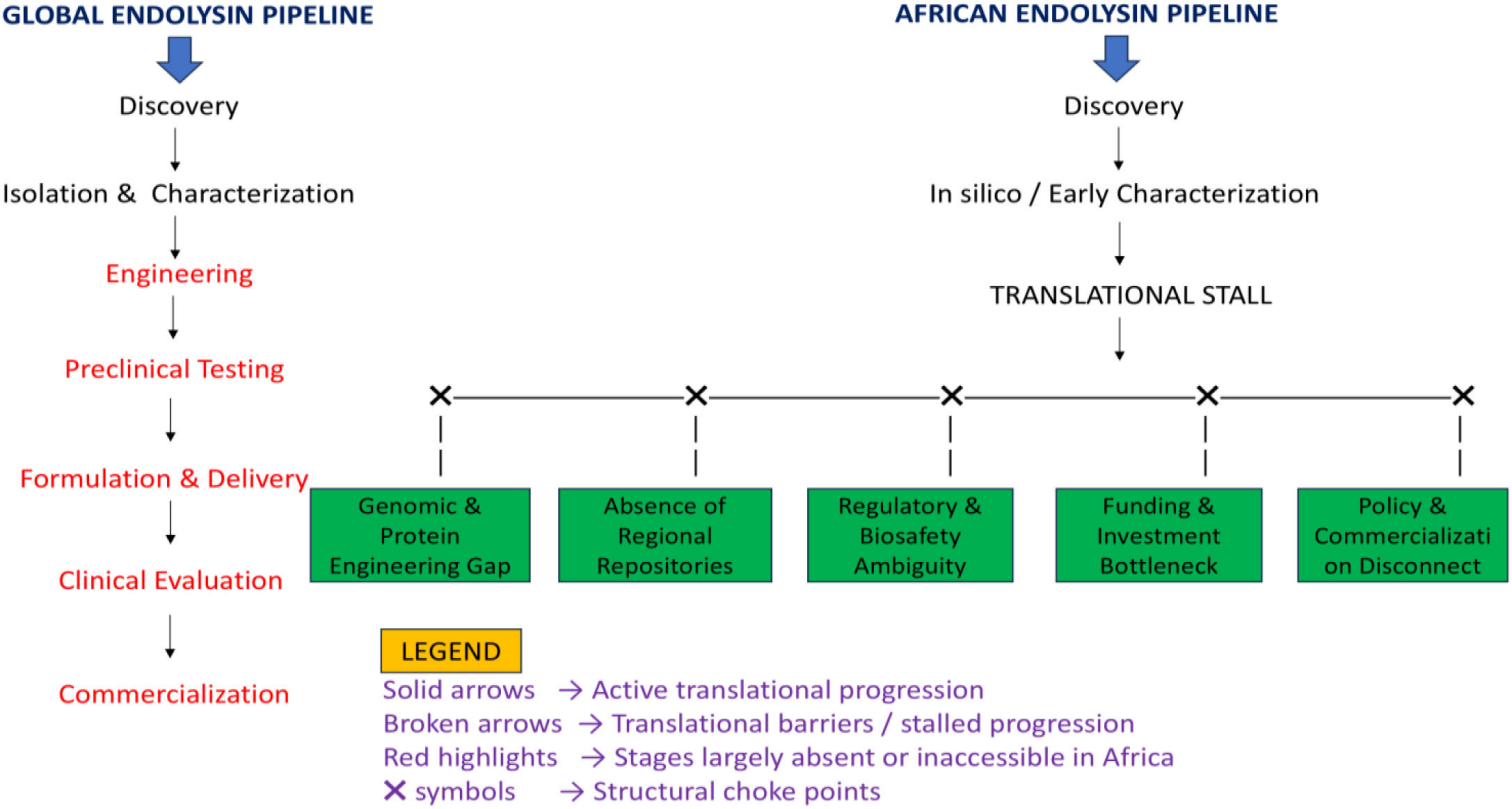
The global endolysin translational pipeline and Africa’s position.

Several interconnected barriers explain why endolysin translation remains limited in Africa. These barriers span scientific capacity, infrastructure, regulation, funding, and policy integration. This section critically examines these barriers thematically, emphasizing how they collectively produce a persistent translational bottleneck (**Figure 1**).

### 3.1 Scientific and technological constraints

#### 3.1.1 Limited local capacity for genomic sequencing, protein engineering, and phage characterization

A major obstacle to advancing endolysins in Africa is the scarcity of advanced infrastructure for high-throughput whole-genome sequencing, metagenomic analysis, bioinformatics, structural modeling, and recombinant protein engineering (Tesema, 2025; Sammut, 2021). Only a few institutions have reliable sequencing, proteomics, or synthetic biology capabilities, restricting most phage research to basic isolation, host-range testing, or in silico lysin gene prediction—rarely progressing to functional validation or expression (Ngene et al., 2023; Aworh et al., 2025; Obidi and Ekpunobi, 2025). Without local access to robust expression, purification, and structural tools, promising genomic leads often remain descriptive and fail to translate into active lysin candidates. Fragmented interdisciplinary collaboration among microbiology, bioinformatics, structural biology, and biotechnology further hinders integrated pipelines, leading to reliance on external partners, limited local ownership, and poor continuity beyond initial studies—unlike the collaborative ecosystems in high-income settings (Leinonen et al., 2019).

#### 3.1.2 Mismatch between global lysin priorities and Africa’s dominant pathogens

Global endolysin efforts predominantly target Gram-positive bacteria like Staphylococcus aureus and Streptococcus spp., driven by disease burdens, funding, and regulatory familiarity in wealthier regions (Liu et al., 2023). In contrast, Africa bears a heavier burden from multidrug-resistant Gram-negative and zoonotic pathogens, including Escherichia coli, Klebsiella pneumoniae, Salmonella spp., Acinetobacter baumannii, and non-typhoidal Salmonella in food systems. Targeting Gram-negatives demands complex outer-membrane engineering strategies, raising technical and cost barriers (Zheng and Zhang, 2024). This misalignment means global lysin advancements often fail to address Africa’s most pressing clinical, agricultural, and environmental AMR challenges (see **Table 1** for infrastructure and target disparities).

**Table 1:** Comparison of global vs. African endolysin research landscape.

Aspect	Global landscape (Europe/North America/Asia)	African landscape
Published studies on endolysins	Hundreds; increasing annually	Limited; largely descriptive or in silico
Engineered lysins in preclinical/clinical stages	Multiple candidates in advanced pipelines	Virtually none locally developed
Locally isolated and sequenced phages	Extensive repositories and databases	Scattered efforts; limited long-term storage
Endolysins tested against regional pathogens	Broad, multi-pathogen panels	Rare; often not Africa-priority pathogens
Regional phage/endolysin repositories	Well-established	Largely absent

### 3.2 Infrastructural and resource dependencies

Beyond scientific capacity, infrastructural limitations significantly impede endolysin translation in Africa. A major gap is the absence of regional phage and endolysin repositories or biobanks capable of long-term storage, standardized characterization, and controlled access to biological materials (Obidi and Ekpunobi, 2025). Such repositories are central to lysin innovation globally, enabling reproducibility, comparative analysis, and cumulative knowledge building.

In African contexts, phage isolates and lysin constructs are frequently stored under short-term laboratory conditions or lost following project completion (Makumi et al., 2021). This discontinuity prevents iterative improvement, cross-study validation, and the establishment of national or regional lysin libraries.

Compounding this challenge is heavy reliance on imported reagents, expression vectors, enzymes, and purification materials. Supply chain delays, currency instability, and high import costs routinely disrupt experimental timelines and inflate research expenses. These constraints disproportionately affect protein-based technologies such as endolysins, which require consistent access to high-quality reagents for expression, purification, and activity testing. Even when funding is available, infrastructural fragility limits scalability and reproducibility, thereby weakening translational momentum.

### 3.3 Regulatory and biosafety challenges

Effective translation of biologic antimicrobials depends on clear and predictable regulatory pathways. In many African countries, regulatory frameworks for phage-derived or enzyme-based antimicrobials remain underdeveloped or ambiguous (Ngene and Umar, 2025). As a result, endolysins often fall into a regulatory grey zone between conventional antibiotics and biologic therapeutics, creating uncertainty regarding classification, approval requirements, and biosafety evaluation.

Importantly, regulatory governance for biomedical products varies substantially across African countries and should not be considered a uniform continental system. National regulatory authorities operate independently and differ in their institutional capacity, technical expertise, and regulatory procedures. For example, the South African Health Products Regulatory Authority (SAHPRA) oversees the evaluation and approval of medicines and biologics in South Africa and maintains relatively advanced regulatory review mechanisms for biopharmaceutical products (Dureja and Dhiman, 2021; Moeti et al., 2022). In Nigeria, the National Agency for Food and Drug Administration and Control (NAFDAC) regulates pharmaceuticals, vaccines, and biologics (WHO, 2024), although regulatory guidance specifically addressing phage-derived therapeutics or recombinant antimicrobial enzymes remains limited. Other national authorities across the continent include the Pharmacy and Poisons Board (PPB) in Kenya (Khaemba et al., 2025), the Food and Drugs Authority (Ghana FDA) in Ghana (Ghana, 2013), the Tanzania Medicines and Medical Devices Authority (TMDA) in Tanzania (Medicines, 2019), the Egyptian Drug Authority (EDA) in Egypt (Mahran, 2023), the Ethiopian Food and Drug Authority (EFDA) in Ethiopia (Shambel, 2024), the National Drug Authority (NDA) in Uganda (Bagonza et al., 2020), the Medicines Control Authority of Zimbabwe (MCAZ) in Zimbabwe (Sithole et al., 2022), and the Pharmacy and Medicines Regulatory Authority (PMRA) in Malawi (Mponda et al., 2025). While these agencies regulate pharmaceuticals and biologics within their respective jurisdictions, many face persistent challenges including limited technical capacity for evaluating complex biologics, insufficient regulatory guidelines for emerging antimicrobial modalities, constrained funding, and limited access to specialized scientific review expertise.

At the continental level, the African Medicines Agency (AMA) has been established to promote regulatory harmonization and strengthen medicine regulation across African Union member states (Abdulwahab et al., 2024). Complementary regional initiatives, such as the African Medicines Regulatory Harmonization Initiative (AMRH) (Ndomondo-Sigonda et al., 2018) and the African Vaccine Regulatory Forum (AVAREF) (Akanmori et al., 2025), seek to improve regulatory collaboration, facilitate joint product assessments, and strengthen institutional capacity across national regulatory systems. Although these initiatives represent important steps toward regulatory convergence, their full operational integration across countries is still evolving.

In addition to regulatory uncertainty, limitations in biosafety level (BSL) infrastructure restrict the ability to conduct advanced pathogenicity testing, animal studies, and containment-based evaluations. High-containment laboratory facilities required for certain microbiological and recombinant protein studies are unevenly distributed across the continent, with stronger infrastructure typically concentrated in a small number of research-intensive countries. Although ethical review systems are improving across many African institutions, specific guidance for genetically engineered antimicrobials and recombinant phage-derived proteins remains limited (Makumi et al., 2021). These regulatory and biosafety gaps collectively slow translational progress, create uncertainty for developers, and discourage investment in the clinical advancement of phage-derived antimicrobial technologies.

### 3.4 Economic and funding limitations

Economic constraints also contribute significantly to the slow translation of endolysin technologies in Africa (Murray et al., 2021). Biotechnology research funding across the continent remains limited and often fragmented, with many programs relying heavily on external donor support. As a result, most projects focus on short-term academic outputs rather than sustained translational development.

In addition, private-sector investment in antimicrobial biotechnology is minimal due to perceived regulatory uncertainty, limited commercialization pathways, and underdeveloped manufacturing infrastructure. These financial constraints frequently prevent promising endolysin research from progressing beyond early proof-of-concept stages.

### 3.5 Policy and systemic integration gaps

At the policy level, phage endolysins remain largely absent from national AMR action plans, One Health frameworks, and innovation strategies across Africa. While many countries have adopted AMR surveillance and stewardship components, few explicitly incorporate next-generation biologics or enzyme-based antimicrobials into strategic planning (Nazir et al., 2023).

Additionally, weak technology transfer systems, limited intellectual property support, and underdeveloped commercialization pathways hinder progression from academic discovery to product development. Without mechanisms for patenting, licensing, and industry engagement, endolysin research remains confined to academic outputs rather than translational deliverables.

These systemic gaps reflect a broader disconnect between scientific research and health innovation governance.

## 4 Africa as a translational bottleneck rather than a knowledge gap

Characterizing Africa’s limited engagement with phage endolysin development as a “knowledge deficit” obscures the structural dynamics that more accurately explain the gap. Across the continent, there is demonstrable expertise in microbiology, bacterial genomics, AMR surveillance, and molecular biology. African laboratories contribute to pathogen sequencing consortia, publish on multidrug-resistant organisms, and increasingly participate in global bioinformatics networks (Nilsson et al., 2025). These competencies constitute the epistemic foundation necessary for endolysin discovery and early-stage characterization.

**Figure 2:**
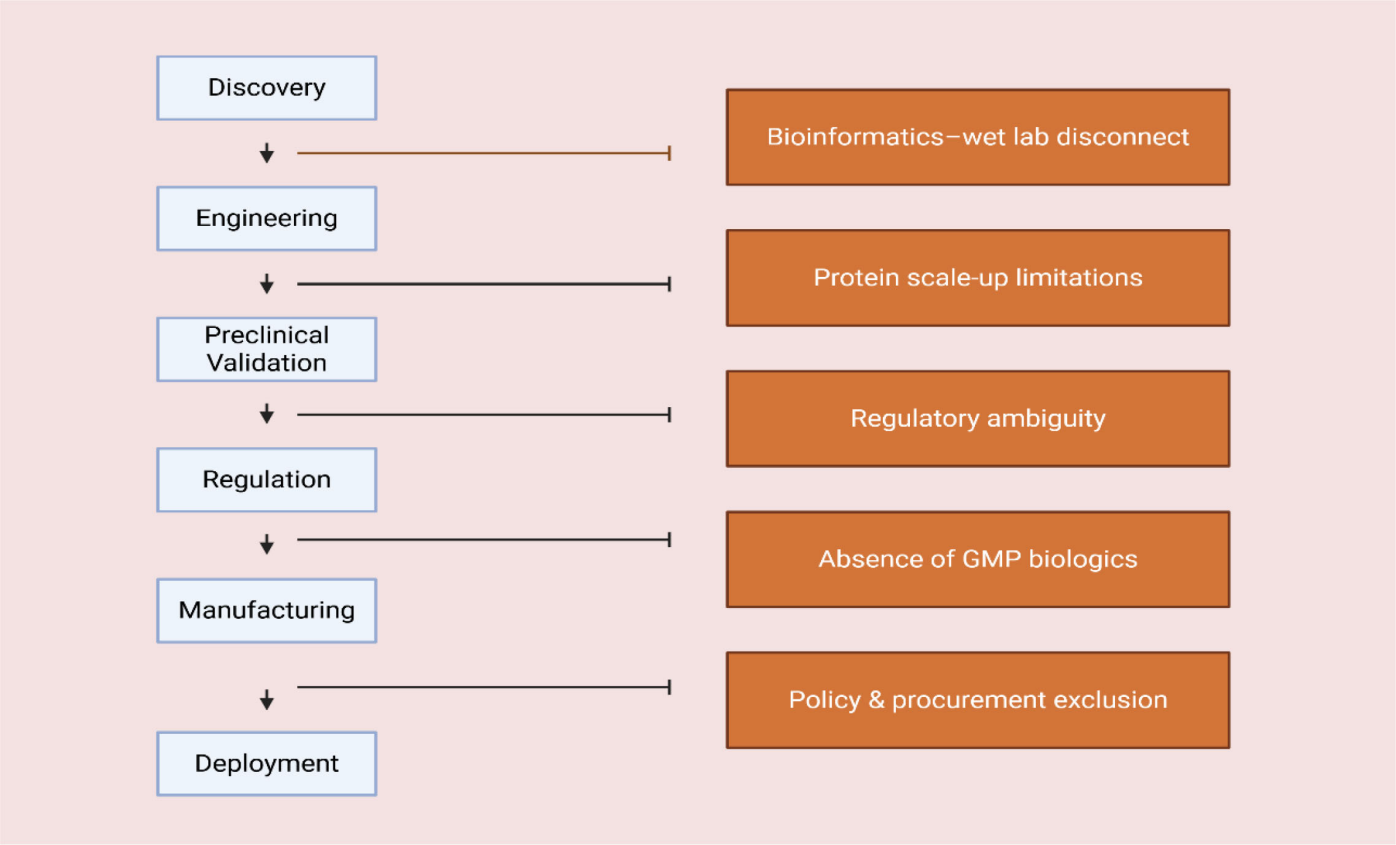
Translational discontinuities across the endolysin innovation pipeline in Africa.

What remains discontinuous is not cognition, but conversion (**Figure 2**). Endolysin innovation requires continuity across institutional layers that do not traditionally operate in synchrony within many African systems. Discovery laboratories must interface with protein engineering platforms; engineered candidates must move into standardized preclinical testing; regulatory authorities must provide predictable classification pathways; manufacturing capacity must accommodate recombinant biologics; and procurement systems must recognize enzyme-based antimicrobials as legitimate tools within AMR strategies. Where these linkages are weak, innovation does not collapse at a single point (it fragments at the interfaces).

In this context, the barriers described above indicate that Africa’s limited engagement with endolysin innovation reflects a systemic translational bottleneck rather than a deficit of scientific knowledge. Bottlenecks differ from gaps. A gap implies missing knowledge that can be remedied through education, training, or exposure. A bottleneck implies systemic constriction: knowledge may enter the pipeline, but structural resistance prevents throughput. This distinction reframes both responsibility and remedy. Training more microbiologists, while valuable, will not by itself produce deployable endolysin therapeutics if regulatory ambiguity, manufacturing absence, and funding discontinuity persist.

Endolysins are especially vulnerable to these fractures because they are biologic antimicrobials situated between established regulatory and industrial categories (Nazir et al., 2023). Unlike small-molecule antibiotics, whose translational pathways are historically institutionalized, or live phage therapy, which can sometimes leverage compassionate-use frameworks, recombinant lysins require coordinated engagement across biotechnology, regulatory science, and industrial protein production. In systems where these domains are institutionally siloed, translation stalls despite scientific plausibility.

Thus, the African endolysin gap should be understood as a systems-integration failure. Discovery occurs. Engineering is conceivable. Policy discourse on AMR exists. Yet the connective tissue between these domains remains thin. Arrested translation (not failed science) best captures the condition.

Recognizing this bottleneck clarifies the intervention strategy. Structural reform must prioritize interface strengthening: formalized discovery-to-engineering pipelines, early regulatory consultation mechanisms, shared protein production facilities, and explicit integration of enzyme-based antimicrobials into AMR and One Health policy architectures. Without such reforms, incremental scientific advances will continue to accumulate without yielding deployable outcomes.

Although endolysins have gained global recognition as next-generation antimicrobials, research activity across Africa remains limited. Most African investigations remain centered on bacteriophage isolation (Ngene et al., 2025), with fewer extending into molecular lysin analysis or recombinant engineering. Additionally, many phages isolated in African studies remain genetically uncharacterized due to limited access to whole-genome sequencing technologies (Ngene et al., 2025).

Regional collaborations have begun to emerge (such as partnerships with PhageAfrica (former Africa Phage Forum), with international phage centers and biotechnology programs) but progress is often hindered by insufficient laboratory infrastructure, high costs of molecular assays, restricted access to reagents, and inadequate funding for AMR-related biotechnology research. The scarcity of trained personnel in genomics, protein engineering, and phage biology further limits the pace of lysin-based innovation across the continent (Ngene et al., 2025).

The studies summarized in **Table 2** were selected through a targeted literature survey of databases including PubMed, Scopus, and Google Scholar. The search employed combinations of relevant keywords such as “phage endolysin,” “bacteriophage lysin,” “endolysin Africa,” “phage-derived enzymes,” “recombinant lysin,” and “bacteriophage antimicrobial Africa.” Articles were shortlisted based on the following criteria: (i) the study reported the identification, cloning, expression, characterization, or antimicrobial evaluation of bacteriophage-derived endolysins; (ii) the research was conducted within African institutions or involved bacterial or environmental isolates originating from African settings; (iii) the study included experimental validation such as enzymatic activity assays, antimicrobial testing, or structural characterization; and (iv) the work was published in a peer-reviewed scientific journal. Preference was given to studies demonstrating potential translational relevance to clinical, veterinary, or food safety applications. The table therefore provides a representative overview of the current landscape of phage endolysin research activities across Africa rather than an exhaustive catalogue of all publications.

**Table 2:** Summary of endolysin-related research activity in Africa.

S/N	Country	Purpose	Target bacterium	Host	Source	Analysis type	Endolysin	Key findings	Reference
1	Egypt	Isolation, cloning, expression, and antibacterial assessment of phage endolysin	*Bacillus cereus* ATCC 11778	*Pseudomonas* phage ZCPS1	Environmental isolate/phage genome	In silico genome annotation, PCR, cloning, SDS-PAGE, SEM, lytic assays	LysZC1	LysZC1 successfully cloned and expressed; showed strong lytic activity against *B. cereus*; caused	Abdelrahman et al., 2024
2	South Africa	Exploration of lysogenic bacteriophages and heterologous expression of endolysins as alternative treatments for APEC and other bacteria	*Escherichia coli (Avian Pathogenic E. coli – APEC); other test bacteria*	*E. coli BL21 (DE3), Yarrowia lipolytica Po1h*	Lysogenic strains screened from poultry-related isolates	PCR screening for prophage genes; induction assays (UV, heat, mitomycin C); bacterial & yeast expression systems; SDS-PAGE; LC-MS/MS; antibacterial assays	Lambda phage endolysin (heterologously expressed)	Successful cloning and expression of lambda endolysin in bacterial systems; proteins confirmed by LC-MS/MS; limited antibacterial effect in assays even with permeabilizing agents; yeast expression confirmed by PCR but low yield; inducible prophage detected carrying *cro* and *cI* genes; study suggests further optimization required for efficacy	Lee, 2018
3	Egypt	Cloning, structural characterization, and evaluation of antibacterial activity of LysZC1	*Bacillus cereus*; also tested on multiple Gram-negatives (*E. coli, Salmonella Typhimurium, Shigella sonnei, Pseudomonas aeruginosa, Klebsiella pneumoniae*)	*Pseudomonas* phage ZCPS1	Environmental phage isolate (ZCPS1)	In silico genome annotation, structural modeling, MD simulation, cloning, expression, purification, biochemical assays, SEM, turbidity reduction, MIC determination	LysZC1	Thermostable (active up to 100 °C), broad-spectrum muramidase with strong lytic activity; highest potency against *B. cereus*; synergistic activity with EDTA against Gram-negatives; stable after 11 months at 4 °C	Abdelrahman et al., 2023

Although bacteriophage endolysins have gained global attention as promising alternatives to antibiotics, very few studies have been conducted within Africa. The research summarized in **Table 2** represents *some of the only documented endolysin-focused investigations from African institutions* as sourced from Google Scholar, Scopus, PubMed, and Research Gate,.

Importantly, despite these emerging studies, no published evidence currently demonstrates a complete translational pathway for phage endolysin development originating from African research institutions. Specifically, there are no documented cases in which an endolysin candidate discovered within Africa has progressed from molecular characterization to intellectual property protection and subsequent preclinical therapeutic evaluation. Existing studies remain largely limited to phage isolation, genomic identification of lysin genes, or preliminary cloning and biochemical characterization. This absence of full translational progression highlights the structural barriers described throughout this review and underscores the need for stronger institutional support systems capable of advancing promising lysin candidates beyond early-stage research.

## 5 Endolysins and Africa’s one health AMR reality

The increasing global burden of antimicrobial resistance has reinforced the importance of the One Health approach, which recognizes the interconnectedness of human, animal, and environmental health (Aslam et al., 2021; Al-Khalaifah et al., 2025). The World Health Organization, Food and Agriculture Organization, and World Organisation for Animal Health jointly advocate for coordinated strategies that address AMR across these domains (World Health Organization, UNEP United Nations Environment Programme, & World Organisation for Animal Health, 2023). Within this framework, phage-derived endolysins represent promising antimicrobial tools capable of targeting bacterial pathogens in multiple sectors simultaneously.

In human health, endolysins offer targeted antimicrobial activity against clinically important pathogens such as *Staphylococcus aureus*, *Streptococcus pneumoniae*, and *Clostridioides difficile* (Mondal et al., 2020; Liu et al., 2023). Their rapid bacteriolytic activity, specificity toward target pathogens, and reduced disruption of beneficial microbiota distinguish them from conventional broad-spectrum antibiotics. These properties make endolysins attractive candidates for treating multidrug-resistant infections and for use in combination therapies that enhance antibiotic efficacy while potentially reducing resistance selection pressure.

Beyond clinical medicine, livestock production systems represent another critical domain within the One Health framework. The extensive use of antibiotics in poultry, cattle, and aquaculture contributes significantly to the emergence and dissemination of resistant bacterial strains (Ngene et al., 2020). Phage-derived endolysins could serve as alternatives or adjuncts to conventional antibiotics in veterinary medicine by targeting key pathogens such as *Salmonella*, *Escherichia coli*, and *Staphylococcus aureus* associated with animal infections (Golban et al., 2025). Their application as feed additives, topical treatments for mastitis, or environmental disinfectants in animal housing facilities could help reduce antimicrobial use while improving animal health and productivity.

Food safety also represents an important application area for endolysins. Bacterial contamination during food processing and storage remains a major source of foodborne disease worldwide (Abebe et al., 2020). Because of their specificity and rapid bactericidal activity, endolysins can be used as biocontrol agents in food systems, particularly for controlling pathogens such as *Listeria monocytogenes* in dairy and ready-to-eat foods (Lee et al., 2023; Grigore-Gurgu et al., 2024). Their ability to selectively eliminate pathogenic bacteria without significantly altering beneficial microbial communities offers advantages over chemical preservatives or broad-spectrum antimicrobials.

Environmental reservoirs of antimicrobial resistance further highlight the relevance of the One Health perspective. Wastewater systems, agricultural runoff, and hospital effluents often contain antibiotic residues and resistant bacteria that facilitate the spread of resistance genes (Evoung Chandja et al., 2024). Endolysin-based interventions could potentially be applied in environmental sanitation strategies, including surface decontamination and wastewater treatment systems, to reduce pathogen loads and limit environmental dissemination of resistant organisms (Rahman et al., 2021).

In the African context, adopting a One Health framework for endolysin development may offer several strategic advantages. Many African countries face interconnected challenges involving human infectious diseases, livestock health, food safety, and environmental sanitation, all of which contribute to AMR emergence and transmission (Ikhimiukor et al., 2022). Integrating lysin-based technologies into existing One Health initiatives coordinated by organizations such as the Africa Centres for Disease Control and Prevention and regional public health institutions could facilitate cross-sector collaboration and accelerate translational research efforts.

Furthermore, the development of lysin-based antimicrobials aligned with One Health principles may provide opportunities for locally relevant innovation, particularly in areas such as livestock disease control, food safety interventions, and community-level infection prevention. Strengthening interdisciplinary collaboration between microbiologists, veterinarians, public health experts, and environmental scientists will therefore be essential for translating laboratory discoveries into practical One Health solutions capable of addressing the complex AMR challenges facing the continent.

### 5.1 Misalignment between global development trajectories and African epidemiology

The pathogens prioritized in global endolysin development pipelines reflect the disease ecology, funding priorities, and regulatory familiarity of high-income regions. This has resulted in a concentration of lysin candidates targeting Gram-positive bacteria responsible for bloodstream, respiratory, and skin infections (Vázquez et al., 2018; Ho et al., 2022; Zhydzetski et al., 2024). While clinically important, this focus does not map cleanly onto Africa’s AMR burden, which is dominated by Gram-negative, zoonotic, and environmentally persistent organisms.

This misalignment has two translational consequences. First, Africa is positioned as a downstream recipient of technologies that may not address its most pressing AMR threats. Second, local research initiatives attempting to realign lysin discovery toward regional pathogens face steeper technical and financial barriers, particularly when engineering is required to overcome structural defenses such as the Gram-negative outer membrane.

### 5.2 Endolysins beyond human medicine

A defining feature of Africa’s AMR challenge is the prominence of non-clinical reservoirs. Antimicrobial use in livestock, aquaculture, and crop production is frequently unregulated, and waste management infrastructures are often inadequate to contain resistant organisms (Ngene et al., 2021; Odey et al., 2024). Endolysins possess characteristics that are uniquely suited to these contexts, including species specificity, environmental biodegradability, and reduced selective pressure for resistance.

Despite this theoretical fit, translation into agricultural or environmental applications is almost entirely absent. This absence reflects regulatory blind spots rather than scientific infeasibility. Most national frameworks lack provisions for enzyme-based antimicrobials outside human medicine, effectively excluding endolysins from One Health intervention portfolios.

## 6 Structural analysis of translational failure points

Translational failure in endolysin development does not occur uniformly across the innovation pipeline; rather, it emerges at specific points where continuity between stages breaks down. These breakdowns are best understood not as failures of individual stages, but as failures of alignment at the interfaces connecting discovery, engineering, validation, regulation, and deployment. To make these interface-specific constraints explicit, **Table 3** maps the dominant translational barriers encountered at each transition point in the endolysin development pathway, highlighting where otherwise successful scientific outputs fail to progress due to structural misalignment rather than technical inadequacy.

**Table 3:** Interface-specific translational barriers affecting endolysin development in Africa.

Translational interface	Functional alignment required	Primary enabling inputs	Dominant failure mode
Discovery → Engineering	Computational–experimental integration	Validated gene candidates, expression-ready constructs	Bioinformatic outputs remain in silico without functional expression
Engineering → Preclinical	Protein production and model continuity	Scalable expression systems, purification platforms, relevant test models	Fragmented expression and purification capacity prevents downstream validation
Preclinical → Regulation	Standardized safety and efficacy translation	Harmonized toxicology, dosing, and efficacy metrics	Regulatory classification ambiguity for recombinant enzymes
Regulation → Manufacturing	Regulatory–industrial coordination	Defined GMP pathways, industry partnerships	Absence of local biologics manufacturing and GMP-certified facilities
Manufacturing → Deployment	Policy–market alignment	Procurement frameworks, AMR and One Health inclusion	Exclusion of enzyme-based antimicrobials from policy and procurement systems

### 6.1 Interface failure rather than stage failure

A recurring pattern in African endolysin research is successful completion of isolated innovation stages without progression to the next. Genes are identified but not expressed; proteins are expressed but not validated in relevant models; proof-of-concept data are generated but not aligned with regulatory expectations. These patterns indicate interface failure, where outputs from one stage are not formatted, supported, or recognized as valid inputs for the next.

Unlike antibiotics, whose translational pathways are well institutionalized, endolysins fall between existing categories (Sabur et al., 2025). This ambiguity amplifies interface fragility, particularly in systems already constrained by limited coordination and resource continuity.

Importantly, these failure modes intensify rather than resolve as endolysin candidates advance along the pipeline, underscoring that translational blockage in African contexts is cumulative and interface-driven rather than stage-specific.

## 7 Regulatory ambiguity as a central translational constraint

Regulatory classification functions as the gatekeeping interface between scientific innovation and real-world deployment (Maccaro et al., 2024). For biologic antimicrobials such as phage endolysins, clarity at this interface determines whether laboratory discoveries progress toward clinical validation or remain confined to experimental research. Although several African regulatory authorities (including the South African Health Products Regulatory Authority, National Agency for Food and Drug Administration and Control, and other national medicines agencies) oversee pharmaceutical and biologic products, regulatory pathways specifically designed for phage-derived enzymes remain largely undefined. As a result, endolysins frequently fall between existing regulatory categories developed for small-molecule antibiotics on one hand and live biological therapeutics on the other (Murray et al., 2021).

This intermediate status creates procedural uncertainty across multiple stages of translational development, including biosafety evaluation, clinical trial authorization, and manufacturing approval. Developers may face uncertainty regarding product classification, regulatory data requirements, and applicable biosafety standards (WHO, 2020). Such ambiguity can delay early regulatory engagement and complicate the design of development pipelines, particularly for academic laboratories and early-stage biotechnology initiatives that lack dedicated regulatory expertise.

**Figure 3:**
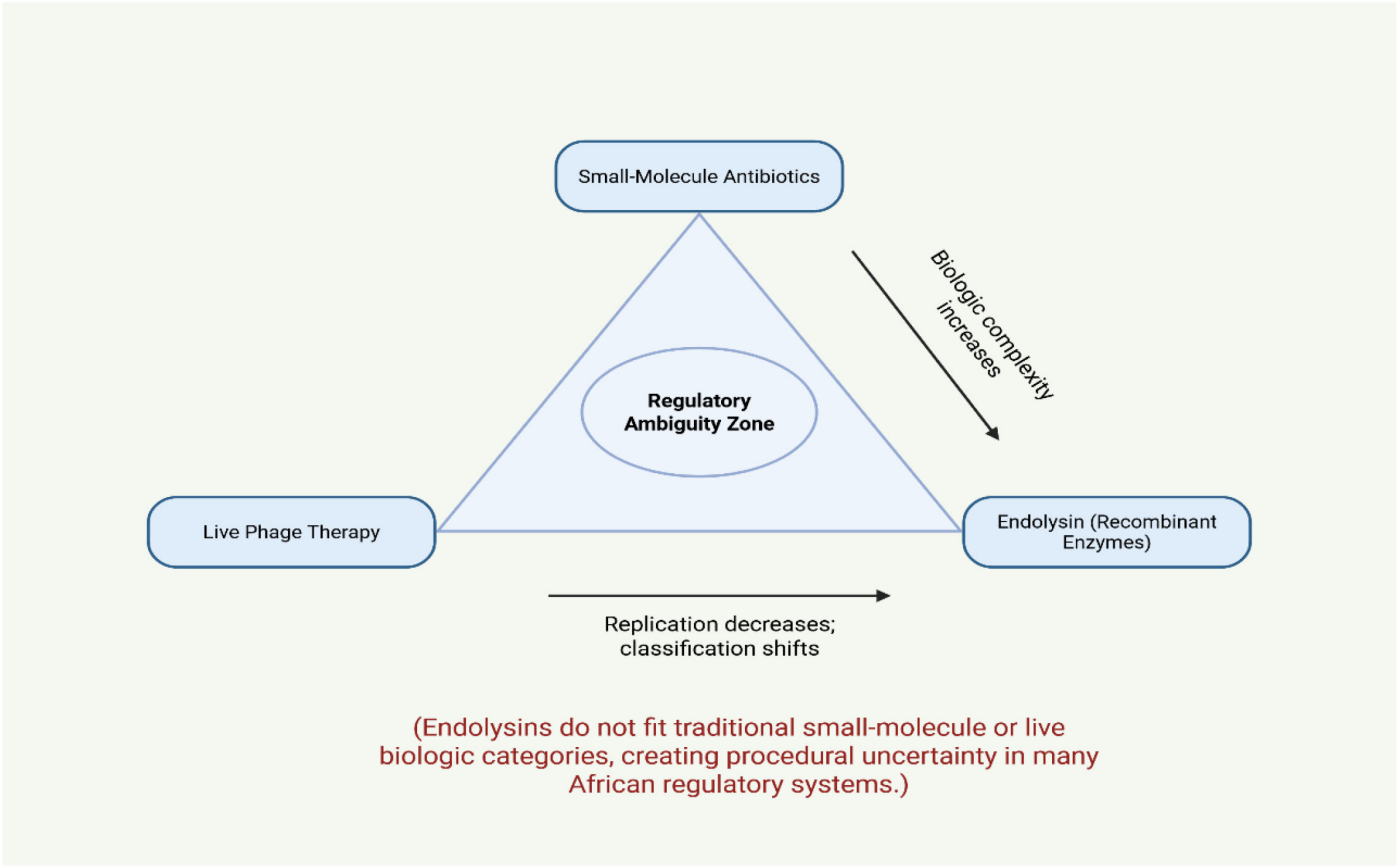
Regulatory positioning of endolysins relative to antibiotics and phage therapy. ¹Small-Molecule Antibiotics (Chemically synthesized entities, Established GMP manufacturing pathways, Standardized clinical trial frameworks, Long-standing pharmacovigilance systems). ²Live Phage Therapy (Replicating biological agents, Adaptive *in vivo* behavior, Compassionate-use precedents in some jurisdictions, Specialized but defined oversight mechanisms). ³Endolysins (Recombinant Enzymes) (Non-replicating biologic therapeutics, Recombinant protein products, Enzymatic bacteriolytic mechanism, Potential application across clinical, veterinary, and environmental sectors, Often lacking explicit regulatory classification).

**Figure 3** conceptually illustrates this regulatory grey zone, highlighting how the hybrid biological nature of endolysins complicates their placement within existing medicinal product frameworks. The resulting uncertainty does not merely delay approval timelines; it can also discourage investment, limit public–private partnerships and reduce incentives for translational research in emerging antimicrobial technologies.

Efforts to address these structural barriers are emerging at the continental level. Initiatives such as the African Medicines Agency, supported by collaborative mechanisms including the African Medicines Regulatory Harmonization Initiative and the African Vaccine Regulatory Forum, aim to strengthen regulatory capacity and encourage coordinated evaluation of medical products across African Union member states (Microbiome Therapeutics Innovation Group and Barberio, 2024). While these frameworks represent important steps toward regulatory convergence, explicit classification guidelines and development pathways for recombinant antimicrobial enzymes have yet to be fully established.

Until such regulatory clarity emerges, this ambiguity will continue to shape the translational landscape for phage endolysins. Addressing regulatory classification early in the development pipeline (through dialogue between researchers, regulators, and policy stakeholders) will therefore be essential for enabling the progression of endolysin technologies from experimental discovery to clinical and public-health application.

### 7.1 Endolysins in a regulatory grey zone

Endolysins do not fit neatly into regulatory categories designed for either small-molecule antibiotics or live biological agents (Murray et al., 2021; Aitken et al., 2025). In African regulatory systems, this ambiguity often translates into procedural paralysis. Without explicit classification, developers cannot anticipate data requirements, timelines, or approval pathways. Regulators, in turn, lack precedent for evaluation, increasing institutional risk aversion.

This uncertainty has a chilling effect on investment and discourages early engagement between researchers and regulatory authorities. In contrast, regions with established biologics frameworks provide clearer translational trajectories even for novel antimicrobial modalities.

### 7.2 Biosafety and ethical review limitations

Advanced endolysin development requires biosafety infrastructure capable of supporting pathogenicity testing, animal studies, and recombinant protein handling. While ethical review capacity has expanded across Africa (Chaudhry et al., 2022; Orievulu et al., 2024), guidance specific to engineered antimicrobial enzymes remains limited. This gap introduces delays and inconsistencies that further weaken translational momentum.

## 8 Economic and innovation-system constraints

### 8.1 Funding models that disincentivize translation

Most African research funding mechanisms prioritize short-term academic outputs over long-term product development (Tijssen and Winnink, 2022; Treptow, 2024). Endolysin translation, however, requires sustained investment across multiple stages with delayed returns. The mismatch between funding structure and translational timelines results in premature project termination despite technical viability.

Private-sector engagement remains minimal due to perceived regulatory risk, uncertain market pathways, and limited manufacturing infrastructure (Mawejje, 2024; Aghabarari et al., 2025). Without mechanisms to de-risk early development, endolysins struggle to attract the capital necessary for advancement.

### 8.2 Weak technology transfer and IP support

Even when promising endolysin candidates emerge, weak intellectual property (IP) frameworks and underdeveloped technology transfer systems significantly constrain progression toward commercialization. In many African research institutions, patenting, licensing, and spin-out formation remain peripheral activities rather than integrated components of research strategy, academic evaluation, or funding incentives (Evans, 2008). As a result, potentially valuable biologic innovations are frequently disseminated through publications without parallel protection or commercialization planning, effectively confining them within academic domains.

This institutional marginalization of technology transfer has several translational consequences (Igodo, 2025; Mukwevho, 2025). First, the absence of early-stage IP protection reduces the attractiveness of endolysin candidates to private-sector partners, who typically require clear ownership, freedom-to-operate assessments, and defined licensing pathways before committing development capital. Without these assurances, even technically promising lysins are perceived as high-risk assets, discouraging industry engagement at precisely the stages where translational momentum must be sustained.

Second, limited capacity within technology transfer offices (TTOs)—including shortages of specialized legal expertise in biologics, enzyme-based therapeutics, and regulatory science—undermines the ability to navigate complex patent landscapes. Endolysins, as recombinant enzymes derived from bacteriophages, often intersect with prior patents on expression systems, engineering strategies, and therapeutic applications. In settings where TTOs lack the resources to conduct robust patent landscaping or negotiate licensing terms, researchers are left without viable pathways to protect or advance their innovations.

Third, weak integration between research institutions, incubators, and industrial partners further isolates academic discoveries from downstream development. Unlike ecosystems where translational research is embedded within innovation clusters, African endolysin research typically occurs in environments with limited access to biomanufacturing expertise, regulatory consultants, or venture development support. This fragmentation reinforces a pattern in which endolysin projects terminate at proof-of-concept stages—not due to scientific failure, but because institutional mechanisms for transition are absent.

Collectively, these constraints produce what can be described as academic containment of innovation, wherein endolysin research generates scholarly outputs but rarely progresses toward protected, scalable, or deployable products. Without deliberate reform (such as strengthening biologics-focused TTO capacity, embedding IP planning into early research design, and aligning academic incentives with translational outcomes), endolysins are likely to remain scientifically promising yet institutionally stranded within African innovation systems.

## 9 Strategic pathways for enabling endolysin translation in Africa

**Figure 4:**
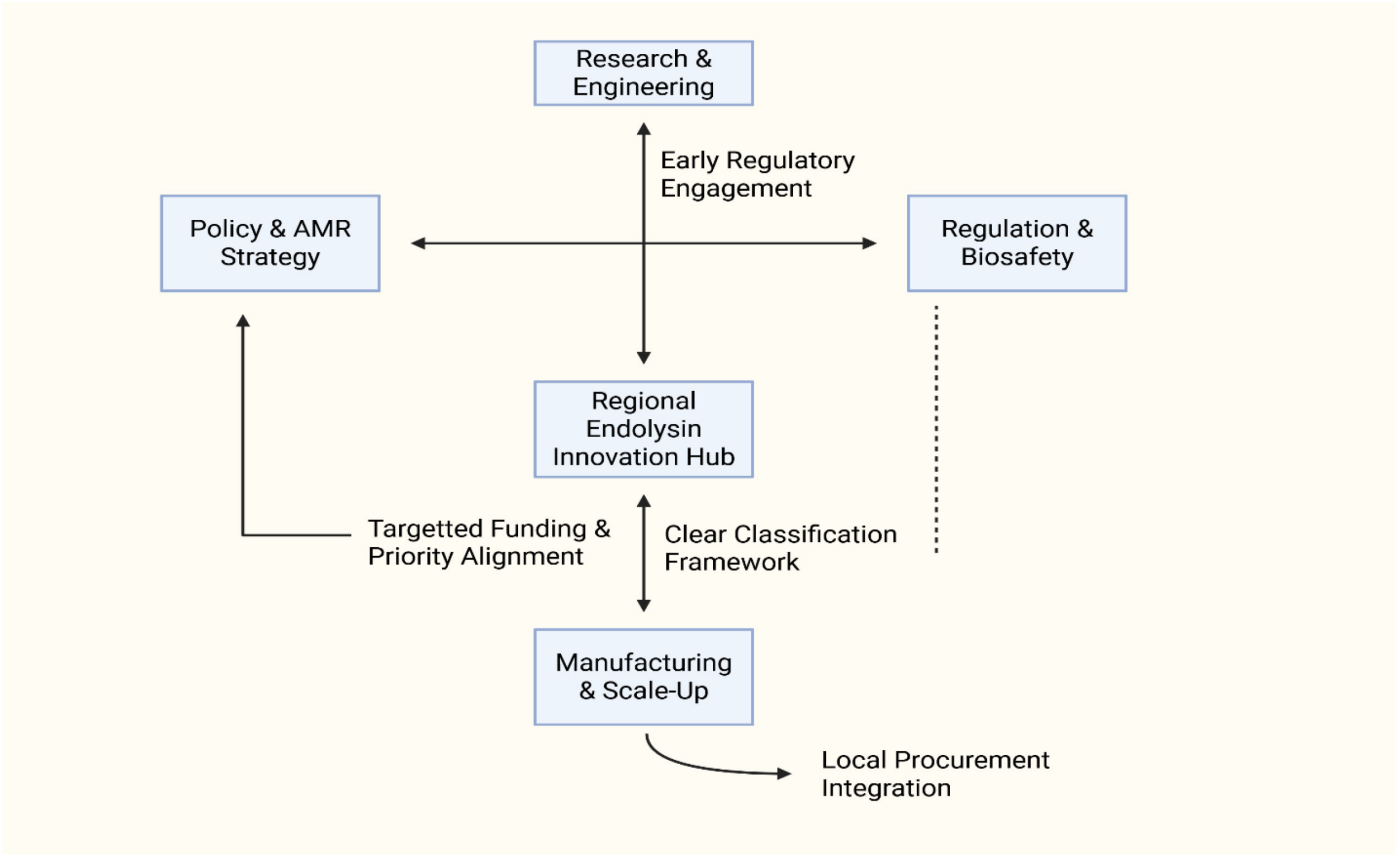
Strategic intervention points for restoring endolysin translational continuity in Africa.

Addressing the translational stagnation of phage endolysins in Africa requires coordinated, systems-level interventions rather than incremental improvements at isolated stages of the innovation pipeline. Because the dominant constraints identified in this review arise at institutional interfaces (between discovery and engineering, regulation and manufacturing, and policy and deployment), effective solutions must be designed to restore continuity across these boundaries. The strategic pathways outlined below target precisely these interface failures, emphasizing shared infrastructure, regulatory predictability, and market creation as complementary levers for translational reform (**Figure 4**).

### 9.1 Regional endolysin innovation hubs

Rather than diffuse and duplicative capacity-building efforts, the establishment of regional endolysin innovation hubs could substantially improve translational efficiency by concentrating complementary expertise and infrastructure within shared platforms (Nazir et al., 2023; Tesema et al., 2025). Such hubs would integrate genomic surveillance, phage and lysin discovery, protein engineering, preclinical validation, regulatory science, and pilot-scale biomanufacturing within coordinated institutional ecosystems. By reducing fragmentation, these hubs would directly address the discovery–engineering and engineering–preclinical interface failures identified earlier in this review.

Critically, regional hubs should prioritize pathogens of regional relevance across human, animal, and environmental domains, aligning innovation agendas with Africa’s epidemiological realities rather than global development trends. Functioning as shared infrastructure rather than institution-specific assets, these hubs could support multi-country collaboration, standardized protocols, and cumulative knowledge building. In doing so, they would transform endolysin research from episodic, project-based activity into sustained translational pipelines.

### 9.2 Regulatory harmonization and early engagement

Regulatory fragmentation across African jurisdictions remains a significant barrier to the translational advancement of emerging biologic antimicrobials. While national medicines regulatory authorities operate under distinct legislative and institutional frameworks, the absence of coordinated guidance for recombinant antimicrobial enzymes creates uncertainty for developers seeking to advance endolysin-based products across multiple markets. Addressing this fragmentation through regulatory harmonization initiatives could substantially reduce development complexity by clarifying classification principles, biosafety expectations, and evidentiary standards for enzyme-based antimicrobials (Magrelli et al., 2025).

Regional coordination mechanisms offer a potential pathway toward such alignment. Collaborative regulatory initiatives at the continental and regional levels are increasingly designed to strengthen regulatory capacity, facilitate joint assessments, and promote convergence in evaluation standards for medical products (Mathieu et al., 2021). By supporting shared technical expertise and coordinated review processes, these mechanisms could help establish clearer approval pathways for novel biologic therapeutics, including recombinant antimicrobial enzymes.

Early regulatory engagement is particularly important for endolysin development (Pohane and Jain, 2015; Zhong and Shang, 2025). Structured dialogue between researchers, regulatory authorities, and industry stakeholders during the preclinical stage can ensure that experimental design, safety assessment, and manufacturing strategies align with anticipated regulatory expectations. Such engagement reduces the risk of late-stage regulatory misalignment and supports more efficient translation from laboratory discovery to applied development.

Importantly, harmonized regulatory frameworks should remain sufficiently flexible to accommodate diverse application contexts. Endolysins possess potential uses beyond human clinical therapeutics, including veterinary medicine, food production systems, and environmental pathogen control. Recognizing these cross-sectoral applications within regulatory frameworks would align endolysin development with broader One Health strategies while expanding viable pathways for translational deployment.

The development of phage-derived endolysin therapeutics in Africa would benefit from integration with existing continental regulatory and public health frameworks. For example, the African Medicines Regulatory Harmonization (AMRH) initiative provides a platform for aligning regulatory standards and accelerating the evaluation of new medical products across regional economic communities. Similarly, the African Union Framework for Antimicrobial Resistance Control, coordinated through the Africa Centres for Disease Control and Prevention (Africa CDC), outlines strategic priorities for surveillance, innovation, and stewardship in combating antimicrobial resistance. Leveraging these structures, regional translational hubs for lysin research could be hosted within established African institutions such as Africa CDC Regional Collaborating Centres, Regional Centres of Regulatory Excellence (RCOREs), and leading research universities with microbiology and biotechnology capacity. Integrating lysin innovation into these policy and regulatory ecosystems could significantly accelerate the translation of laboratory discoveries into clinically deployable antimicrobial solutions across the continent.

### 9.3 Policy integration and market creation

Scientific advancement alone is insufficient to sustain translational momentum in the absence of policy pull. Explicit inclusion of endolysins within national AMR and One Health action plans would signal institutional legitimacy, create demand-side incentives, and integrate enzyme-based antimicrobials into formal intervention portfolios. At present, African endolysin research has largely been driven by investigator-initiated discovery and externally funded proof-of-concept studies (a classic scientific push model) without corresponding mechanisms to generate downstream demand.

The absence of procurement frameworks, reimbursement pathways, and regulatory prioritization has limited translational progression even where scientific feasibility exists (Hoekman and Njinkeu, 2017). **Figure 4** illustrates how targeted policy pull mechanisms (such as public procurement for infection prevention, prioritized regulatory review, and AMR-linked innovation incentives) can restore continuity between scientific development, manufacturing, and deployment. By creating predictable markets, particularly in non-clinical One Health domains, policymakers can de-risk early development and attract private-sector engagement.

Market creation does not require immediate large-scale deployment (De Melo et al., 2020). Pilot procurement programs, demonstration projects in agriculture or healthcare-associated infection control, and integration into AMR stewardship initiatives could provide early validation environments for endolysin technologies. These measures would convert policy intent into operational demand, transforming endolysins from speculative innovations into deployable tools within African AMR strategies.

Systems-level representation of institutional nodes and targeted intervention pathways required to restore translational continuity for endolysin development in African innovation systems. Reinforcing feedback loops emphasizes integration across research, regulation, manufacturing, and policy domains.

## 10 Future outlook: endolysins as a test case for translational equity

Phage endolysins represent more than a promising class of next-generation antimicrobials; they function as a diagnostic lens through which the structural dynamics of innovation systems can be evaluated (Shemyakin et al., 2020; Na, 2024). Their stalled progression within many African contexts does not primarily reflect scientific incapacity, but rather reveals the institutional, regulatory, and infrastructural conditions that determine whether biologic technologies move from laboratory insight to public health utility.

Endolysins sit at the intersection of molecular microbiology, recombinant protein engineering, regulatory science, and biomanufacturing (Lee et al., 2023). As such, they demand a level of cross-sector coordination that exposes systemic weaknesses when they exist. Where innovation systems are vertically integrated (with predictable regulatory pathways, functional technology transfer mechanisms, pilot-scale biologics manufacturing, and aligned AMR policy frameworks) translation proceeds. Where these components operate in isolation, promising technologies remain suspended between discovery and deployment.

In this respect, Africa’s limited engagement with endolysin translation mirrors broader patterns affecting advanced biologic therapeutics, vaccine platforms, genomic diagnostics, and other emerging health technologies. The challenge is not conceptual awareness, nor even early-stage scientific capacity; it is the absence of sustained translational continuity. Endolysins, therefore, serve as a test case for translational equity: if systems can be restructured to support their progression, the same reforms will likely strengthen the continent’s capacity to absorb, adapt, and co-develop future antimicrobial innovations.

Addressing these constraints requires strategic rather than incremental reform. Investment must extend beyond isolated research grants toward the construction of translational infrastructure (regional protein engineering hubs, shared preclinical validation platforms, and pilot biologics manufacturing facilities capable of Good Manufacturing Practice (GMP) compliance). Regulatory authorities require harmonized frameworks that explicitly classify recombinant enzyme-based antimicrobials, reducing ambiguity and encouraging early engagement between developers and oversight bodies (Salwan and Sharma, 2026). Equally critical is the integration of enzyme-based therapeutics into national AMR and One Health strategies, creating policy pull to complement scientific push.

Without such structural recalibration, Africa risks remaining positioned at the terminus of global antimicrobial innovation pipelines (receiving externally developed technologies that may not align with its epidemiological priorities, market structures, or ecological realities). This passive recipient model perpetuates dependency, constrains local problem-solving capacity, and limits the continent’s influence over emerging therapeutic agendas.

Conversely, deliberate investment in translational architecture offers an alternative trajectory. By strengthening institutional interfaces (between discovery and engineering, regulation and manufacturing, policy and procurement), African innovation systems can transition from episodic participation to sustained co-production. In doing so, endolysins could become not merely imported therapeutics but regionally optimized solutions targeting locally dominant pathogens across human, animal, and environmental domains.

## 11 Conclusion

Phage endolysins illuminate a central paradox in Africa’s antimicrobial resistance response: Scientific knowledge is expanding, yet translational throughput remains constrained. This review demonstrates that enabling endolysin translation in Africa requires strengthening regulatory, infrastructural, and innovation-system interfaces.

Reframing the challenge as one of translational equity shifts the focus from deficit narratives to structural reform. The steps required to enable endolysin translation (regulatory clarity, coordinated investment in biologics infrastructure, strengthened technology transfer systems, and One Health policy integration) are the same reforms needed to enhance participation in future antimicrobial innovation cycles.

Failure to act risks entrenching a model in which Africa remains a downstream consumer of externally developed therapeutics, perpetuating technological dependency in the face of a disproportionate AMR burden. Strategic, systems-level investment can instead position the continent as a co-producer of next-generation antimicrobials aligned with its epidemiological realities and development priorities.

Endolysins, therefore, stand not only as potential therapeutic tools, but as a measure of whether translational systems can be reconfigured toward greater inclusion, resilience, and innovation sovereignty.
